# On the robustness of [^18^F]-FDG-PET radiomic features to variations in image acquisition and reconstruction settings: A phantom study

**DOI:** 10.1371/journal.pone.0335219

**Published:** 2025-10-22

**Authors:** Syafiq Ramlee, Maria Delgado-Ortet, Lorena Escudero Sanchez, Luigi Aloj, Roido Manavaki

**Affiliations:** 1 Department of Radiology, University of Cambridge, Cambridge, United Kingdom; 2 MRC Human Genetics Unit, Institute of Genetics and Cancer, University of Edinburgh, Edinburgh, United Kingdom; 3 Cancer Research United Kingdom Cambridge Centre, University of Cambridge, Cambridge, United Kingdom; 4 Department of Radiology, Cambridge University Hospitals NHS Foundation Trust, Cambridge, United Kingdom; Fondazione Policlinico Universitario Agostino Gemelli IRCCS, ITALY

## Abstract

**Background:**

Greater access to clinically meaningful data from [^18^F]-FDG-PET images could be made possible through radiomics. However, the vulnerability of radiomic measurements to changes in image acquisition and reconstruction settings has raised concerns on their reliability in clinical practice.

**Methods:**

Using the NEMA-IQ phantom, we evaluated the robustness of [^18^F]-FDG-PET radiomic features to variations in acquisition duration, reconstruction algorithm, transaxial matrix size, z-axis filtering, Gaussian smoothing, and other reconstruction algorithm-specific settings (number of iterations, subsets, updates, and penalisation factors). Feature robustness was assessed using the coefficient of variation (CV < 10%) and intraclass correlation coefficient (ICC > 0.9). Non-robust features were examined for dependencies on these parameters that could be corrected using simple mathematical equations. Using mixed-effects models, we also explored whether differences in region volume or intensity could explain the variability of feature values.

**Results:**

Our findings demonstrated that the majority of [^18^F]-FDG-PET radiomic features were not robust to variations in image acquisition/reconstruction parameters, with features displaying the least stability to matrix size. Robust features mainly comprised shape-based and entropy-related measurements. Most non-robust features did not possess a dependency on acquisition/reconstruction settings that could be corrected using simple equations. The volume and intensity of interrogated regions were also shown to be likely determinants of feature variability to these settings.

**Conclusions:**

Care should be taken when handling radiomic data extracted from heterogeneously acquired/reconstructed [^18^F]-FDG-PET images. Alternative strategies could be required to mitigate the effects of variations in these parameters on radiomic features.

## 1. Introduction

There is an established belief that radiological images contain clinically useful clues about disease that are invisible to the human eye [1], and interest in deriving more information out of these images is rapidly growing [2]. To this end, radiomics has emerged as a distinct field concerned with converting medical images into objective, quantitative, and mineable data [1,3]. This conversion involves measuring the relationship between groups of two or more image voxels, thereby unlocking textural or higher-order details embedded within these images.

The potential utility of radiomics in oncological applications has been covered extensively in the literature [4,5], including its use for classifying tumour subtypes [6,7], identifying molecular characteristics [8,9], and predicting survival [10,11] and treatment response [12,13]. Radiomic features obtained from [^18^F]-fluorodeoxyglucose positron emission tomography ([^18^F]-FDG-PET) images, in particular, have been correlated with clinical outcomes for a variety of cancer patients [14,15]. These features have also performed better than conventional standardised uptake value (SUV) metrics in predicting patient survival and treatment response [16,17].

Yet, despite their advantages, radiomic measurements can be susceptible to variations in imaging parameters, leading to scepticism surrounding their adoption in the clinic [18]. These parameters include those at the image acquisition and reconstruction level (e.g., acquisition duration per bed, transaxial matrix size, and reconstruction algorithm) – the impact of which on PET radiomic features has been the subject of a number of published investigations, both in patient [19–24] and phantom [21,25–30] studies. However, there is limited research on how radiomic features respond to methods that could potentially mitigate their instability to these parameters, including at conditions devoid of tumour image heterogeneity. This baseline variability is important to assess given that it can influence the ability of radiomic measurements to accurately capture tumour heterogeneity. Moreover, as radiomic features may depend on the volumes and intensities of interrogated regions [26,29,31–33], it remains elusive whether these factors exert a differential effect on the robustness of features. These evaluations may facilitate comparison of radiomics analyses across imaging protocols.

A potential strategy to address feature non-robustness involves normalising feature values with respect to the parameter of interest. This was shown to be beneficial in addressing the instability of radiomic features to variations in voxel size and intensity resolution [33–35]. Additionally, a previous study indicated tumour [^18^F]-FDG-PET radiomic features may exhibit a systematic dependency on image processing parameters which could be modelled and corrected using simple mathematical equations [36]. Such a correction framework could possibly be applied in the context of image acquisition/reconstruction settings, and may offer a software-agnostic way to pool radiomic data from differently acquired/reconstructed images.

In this present study, our objectives were three-fold: (i) to evaluate the robustness of phantom-derived [^18^F]-FDG-PET radiomic features against variations in image acquisition and reconstruction settings; (ii) to explore whether non-robust features exhibit a systematic dependency on these settings that could be mitigated via correction using simple mathematical functions; and (iii) to examine the effect of region volume and intensity on the variability of features. Our study indicated that [^18^F]-FDG-PET radiomic features depend on image acquisition and reconstruction settings in a manner not correctable by simple equations, requiring alternative solutions to ensure reliable radiomics analyses across imaging protocols.

## 2. Materials and methods

**Fig 1:**
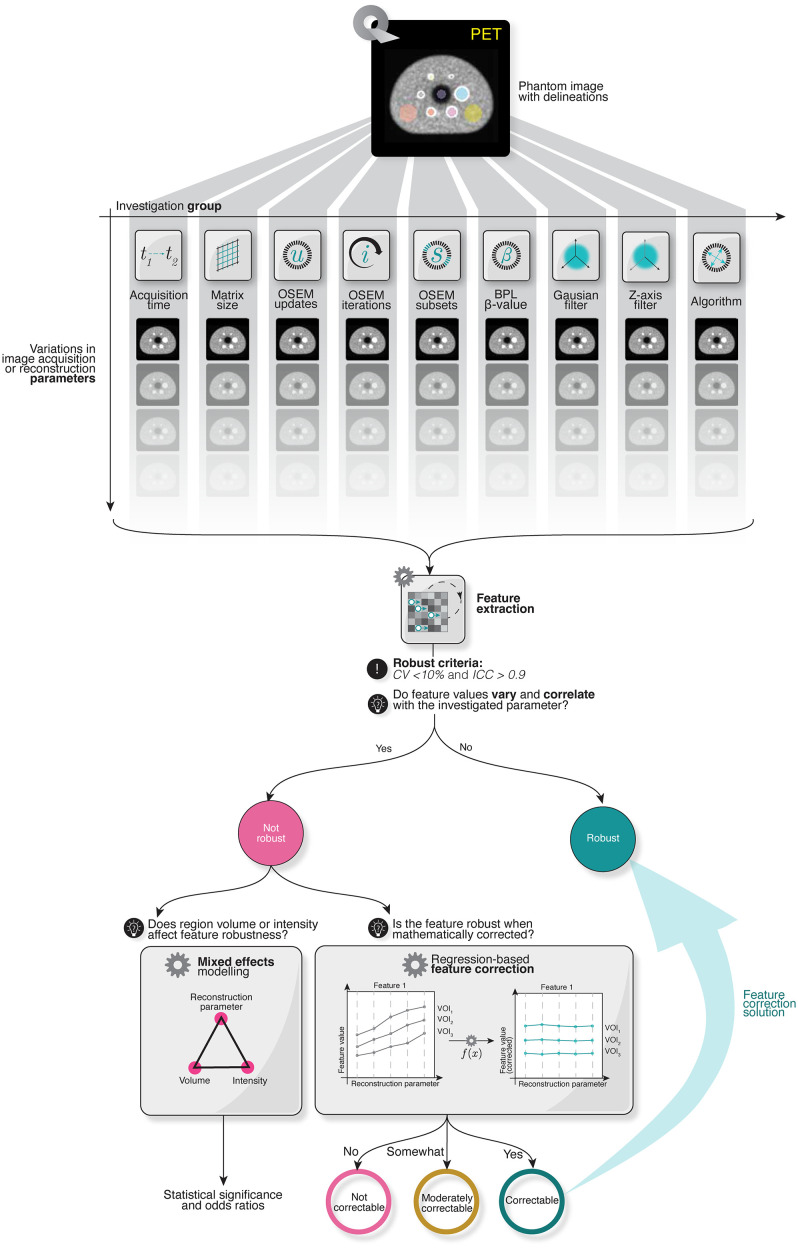
Schematic representation of the workflow of this study.

The design of this study is summarised in Fig 1.

### 2.1. Phantom preparation and image acquisition

**Fig 2:**
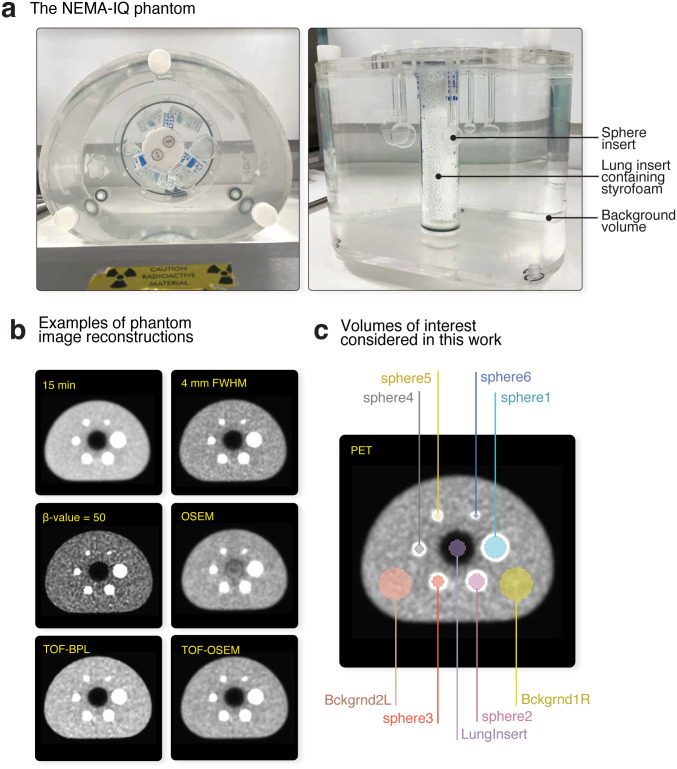
The NEMA-IQ phantom (*NEMA IEC Body Phantom Set*). The main components of the phantom **(a)**, some example [^18^F]-FDG-PET reconstructions **(b)**, and the nine volumes of interest (c) considered in this work.

The anthropomorphic National Electrical Manufacturers Association Image-Quality (NEMA-IQ) phantom (*Data Spectrum Corp*), depicted in Fig 2a, comprises six fillable sphere inserts with internal diameters of 37, 28, 22, 17, 13, and 10 mm, and a cylindrical “lung” insert at the centre of the phantom containing material with a low atomic number (styrofoam). All spheres and the background volume of the phantom were filled with a mixture of [^18^F]-FDG and water at a sphere-to-background ratio of 4:1. At the time of acquisition, the radioactivity concentration in the spheres was approximately 20 kBq/mL. The phantom was scanned over 1 bed position with a transverse field of view of 60 cm for 15 min using a *GE SIGNA* PET/MR scanner (*GE Healthcare*).

### 2.2. Image acquisition and reconstruction parameters

A total of 94 variations in image acquisition/reconstruction settings were considered in this work; a detailed breakdown of the parameters evaluated is furnished in Table 1. These settings were divided into 9 *groups* of investigation, which explored: 6 algorithm choices of either the ordered subsets expectation maximisation (OSEM [*VUE Point, GE Healthcare*]) or Bayesian penalised likelihood (BPL [*Q.Clear, GE Healthcare*]) method, with or without point spread function (PSF) modelling and/or time of flight (TOF) implementation; 11 acquisition durations; 4 transaxial image matrix sizes; 21 post-reconstruction isotropic Gaussian smoothing filter widths; and 4 z-axis filters. For (TOF-)OSEM reconstructions, investigations included 24 combinations for updates (iterations×subsets), 8 for number of iterations, and 3 for number of subsets, while 13 penalisation factors (β-values) were examined for BPL-reconstructed images. For each investigation group, all parameters other than the one being investigated were kept at fixed values to facilitate comparisons, as indicated in Table 1. Corrections for normalisation, dead-time, random events, scatter, sensitivity, and isotopic decay were applied as implemented on the scanner. Attenuation correction was performed using a computed tomography (CT)-based template μ-map of the phantom. Images of some example [^18^F]-FDG-PET reconstructions are provided in Fig 2b.

**Table 1:** List of image acquisition and reconstruction parameters considered in this work.

Investigation group	Parameter variations	Constants
Acquisition time (min/bed)	1, 1.5, 2, 3, 4, 5, 6, 8, 10, 12.5, and 15	Matrix size: 256 × 256 Z-axis filter: None Gaussian filter: 5 mm FWHM Iterations × subsets: 2 × 28 Algorithm: TOF-OSEM
Matrix size	128 × 128, 192 × 192, 256 × 256, and 384 × 384	Acquisition time: 3 min/bed Z-axis filter: None Gaussian filter: 5 mm FWHM Iterations × subsets: 2 × 28 Algorithm: TOF-OSEM
Z-axis filter	None, Light, Standard, and Heavy	Acquisition time: 3 min/bed Matrix size: 256 × 256 Gaussian filter: 5 mm FWHM Iterations × subsets: 2 × 28 Algorithm: TOF-OSEM
Gaussian Filter (mm FWHM)	0, 0.5, 1, 1.5, 2, 2.5, 3, 3.5, 4, 4.5, 5, 5.5, 6, 6.5, 7, 7.5, 8, 8.5, 9, 9.5, and 10	Acquisition time: 3 min/bed Matrix size: 256 × 256 Z-axis filter: None Iterations × subsets: 2 × 28 Algorithm: TOF-OSEM
BPL β-value	50, 100, 150, 200, 250, 300, 350, 400, 500, 600, 700, 800, and 1000	Acquisition time: 3 min/bed Matrix size: 256 × 256 Algorithm: TOF-BPL
OSEM update combinations (iterations × subsets)	1 × 16, 1 × 28, 1 × 32, 2 × 16, 2 × 28, 2 × 32, 3 × 16, 3 × 28, 3 × 32, 4 × 16, 4 × 28, 4 × 32, 5 × 16, 5 × 28, 5 × 32, 6 × 16, 6 × 28, 6 × 32, 8 × 16, 8 × 28, 8 × 32, 10 × 16, 10 × 28, and 10 × 32	Acquisition time: 3 min/bed Matrix size: 256 × 256 Z-axis filter: None Gaussian filter: 5 mm FWHM Algorithm: TOF-OSEM
OSEM iterations	1, 2, 3, 4, 5, 6, 8, and 10	Acquisition time: 3 min/bed Matrix size: 256 × 256 Z-axis filter: None Subsets: 16–32^†^ Gaussian filter: 5 mm FWHM Algorithm: TOF-OSEM
OSEM subsets	16, 28, and 32	Acquisition time: 3 min/bed Matrix size: 256 × 256 Z-axis filter: None Iterations: 1–10^††^ Gaussian filter: 5 mm FWHM Algorithm: TOF-OSEM
Algorithm	OSEM (*VPHD*), TOF-OSEM (*VPFX*), OSEM + PSF (*VPHD-S*), TOF-OSEM + PSF (*VPFX-S*), BPL (*QCHD*), and TOF-BPL (*QCFX*)	Acquisition time: 3 min/bed Matrix size: 256 × 256 Z-axis filter: None Gaussian filter: 0 mm FWHM BPL β-value: 350 Iterations × subsets: 2 × 28

^†^Radiomic feature values across subset schemes were averaged per iteration number, to produce a single value for each feature.

^††^Radiomic feature values across iteration schemes were averaged per subset number, to produce a single value for each feature.

OSEM: ordered subsets expectation maximisation; BPL: Bayesian penalised likelihood; TOF: time-of-flight; PSF: point spread function; VPHD: VUE Point HD; VPFX: VUE Point FX; VPHD-S: VUE Point HD-S; VPFX-S: VUE Point FX-S; QCHD: Q.Clear HD, QCFX: Q.Clear FX, FWHM: full width at half maximum.

### 2.3. Radiomic feature extraction

Nine spherical regions or volumes of interest (VOIs) were manually drawn on a phantom image using ITK-SNAP version 4.2 [37]. The VOIs represented the six active sphere inserts (“sphere1” to “sphere6”, from largest to smallest), two samples of the background compartment (“Bckgrnd1R” and “Bckgrnd2L”), and one sample of the lung insert (“LungInsert”), as exemplified in Fig 2c. These VOIs were subsequently propagated to each reconstructed image to derive radiomic features.

Using the open-source *PyRadiomics* version 3.0.1 package [38], on Python version 3.10.8 [39], we extracted 107 Image Biomarker Standardisation Initiative (IBSI)-compliant radiomic features from the following families for each VOI: shape-based (*n* = 14), first-order statistics (*n* = 18), grey-level co-occurrence matrix (GLCM) (*n* = 24), grey-level dependence matrix (GLDM) (*n* = 14), grey-level run-length matrix (GLRLM) (*n* = 16), grey-level size zone matrix (GLSZM) (*n* = 16), and neighbouring grey-tone dependence matrix (NGTDM) (*n* = 5). GLCM and GLRLM features were computed using the average of corresponding matrices over 13 spatial directions in 3D (26-connectivity), with a single voxel offset for the former. Feature families pre-processed with mathematical filters (higher-order features) were not evaluated in this work. Intensity resolutions were set to a fixed bin number of 64 to keep consistent with [36]. We note that for matrix size investigations, invalid features were obtained for the smallest sphere (“sphere6”) at the smallest matrix size (128 × 128) due to insufficient voxels for radiomic computation, and were therefore excluded from this work.

### 2.4. Feature robustness assessment

Robustness of features was defined as a function of the average of the within-region percentage coefficient of variation across regions (*CV*_*mean*_) per feature, as well as the intraclass correlation coefficient (ICC) from a single source, two-way mixed effects model determining the agreement between measurements. Thresholds of *CV*mean**<10% and ICC > 0.90, as adopted in earlier works [36,40,41] for comparability, established the robust criteria and determined robust features.

### 2.5. Identification of correctable features

Eight regression functions, *f(x),* were fitted to model the mean relationship between non-robust feature values and image acquisition/reconstruction parameters, as implemented in [32,36]. These functions were: *f(x)=α·x + β, f(x)=α·*x**^*2*^* + β, f(x)=α·*x**^*3*^* + β, f(x)=α/x + β, f(x)=α/*x**^*2*^* + β, f(x)=α/*x**^*3*^* + β, f(x)=α·log(x)+β*, and *f(x)=α/log(x)+β* where *α* and *β* in this context are fit parameters respectively, and *x* is the imaging parameter under investigation. Model fits were performed using an iteratively reweighted least squares algorithm with intrinsic weights in the form of the reciprocal of feature variance values computed across regions. The dependencies of feature values on image acquisition/reconstruction parameters were deemed to be best described by the function that had attained the lowest Akaike information criterion (AIC) value amongst the ones tested.

Feature corrections for each region were implemented by using a rearranged form of the best-fitting function, as applied in [32,36], e.g., **f(x)=*α·*log(x)+*β, *f**_*corrected*_**(x)=(f(x)−*β*)/log(x).** For certain groups of investigation, *x* variables were rescaled by an arbitrary factor to circumvent division by zero errors during correction. Specifically, when considering variations in acquisition duration, z-axis filters, and number of iterations, values were multiplied by 10 (e.g., *f(10·x)=α/(10·x)+β*); and when considering Gaussian filter widths, the x-variable was shifted by 2 (e.g., *f(x + 2)=α·(x + 2)+β*). Z-axis filters were mapped from categorical to numerical values using the weights of their respective filtering kernels (“None” mapped to 1, “Light” to 2, “Standard” to 4, and “Heavy” to 6). Corrections based on mathematical equations were not assessed with respect to algorithms given the categorical nature of these variables.

Comparison of CV and ICC values pre- and post-correction was evaluated using the Wilcoxon signed-rank test. Features with a statistically significant reduction in *CV*mean** and improvement in ICC were classified into *correctable* if the robust criteria (*CV*mean,corrected** <10% and ICC*corrected* >0.90) were met, and *moderately correctable* if such criteria remained unsatisfied. Features were otherwise categorised as *not correctable*.

### 2.6. Dependence of feature variability on region volume and intensity

We investigated how region volume and intensity factors may explain, at least in part, the underlying variability of non-robust [^18^F]-FDG-PET feature values across the imaging parameters studied. To this end, volume information and the mean intensity values for each region were determined using the shape-based *MeshVolume* and first-order *Mean* radiomic feature, respectively. Linear mixed-effects models incorporating the reconstruction parameter under investigation, region volume, and region intensity as the fixed effects, and applying per-region random intercepts and slopes, were used to assess the differential response of feature values to these factors. For categorical predictors, only per-feature random intercepts were included. Fixed-effects coefficients and their corresponding *p*-values were extracted for each feature and investigation.

### 2.7. Statistical analysis across features and investigations

Logistic mixed-effects regression with feature-specific random intercepts was employed to summarise findings across families and/or investigations. This approach facilitated calculation of the predicted probabilities of resulting in robust (PP_robustness_) or correctable and moderately correctable (PP_correctability_) outcomes for each family and investigation using:


$$PP=\frac{\text{1}}{\text{1}+e^{-(b+aX)}}$$

where PP is the predicted probability, X is the fixed-effects predictor (feature family, investigation groups), and *a* and *b* are the estimated parameters of the model. PP values were reported as the median with interquartile range (IQR).

Results for the effect of region volume or intensity on feature robustness when compared to the parameter under investigation were reported as odds ratios (OR) with 95% confidence intervals (CI). Differences in results between groups of investigations were assessed using a two-sample test based on the Cramér-von Mises statistic, with *p*-values from post-hoc analyses adjusted for multiple comparisons using the Bonferroni method. Statistical significance was defined as *p* < 0.05. All analyses were conducted in R, version 4.4.0 [42].

## 3. Results

### 3.1. Robust features

**Fig 3:**
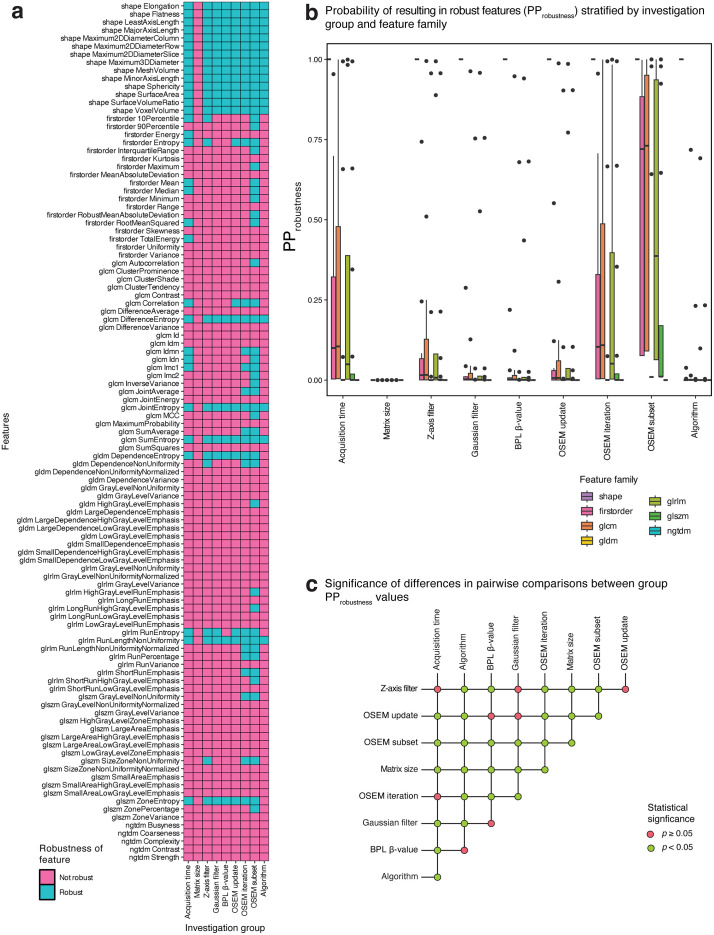
Robustness of radiomic features to variations in image acquisition and reconstruction settings. Feature robustness categorisations (a) and the predicted probabilities of resulting in robust features (PP_robustness_) per feature family **(b)**, both stratified by investigation group. Significance of differences in PP_robustness_ values between investigation groups **(c)**.

Robustness categorisations for each radiomic feature across investigation groups are presented in Fig 3a, with a breakdown of the proportions for each feature family provided in S1 Table. Scatter plots of the response of each robust feature against parameter variations have been deposited in https://github.com/SyafiqRamlee/robust-radiomics-img-recon.

Shape features were unaffected by variations in any of the parameters explored in this work apart from transaxial matrix size which failed to yield any robust features across families. There were also no instances of robust NGTDM features in our analysis. For other families, robust categorisations were sporadic, with a notably low mean proportions of robust features across investigations per family (13% for first-order, 20% for GLCM, 9% for GLDM, 16% for GLRLM, and 9% for GLSZM).

Despite these low figures, robust categorisations were predominantly clustered around entropy or related measures. GLCM *DifferenceEntropy*, GLCM *JointEntropy,* GLCM *SumEntropy*, and GLRLM *RunLengthNonUniformity* were robust to variations in any investigated parameter barring transaxial matrix size. Other entropy-related features (first-order *Entropy*, GLRLM *RunEntropy*, GLDM *DependenceEntropy*, and GLSZM *ZoneEntropy*) achieved robustness in more than half of investigation groups.

Fig 3b depicts the predicted probabilities of achieving feature robustness (PP_robustness_) for each investigation group. Results from pairwise comparisons between groups using the two-samples test based on the Cramér-von Mises statistic are presented in S2 Table and Fig 3c.

We found that [^18^F]-FDG-PET radiomic features were the least affected by the number of OSEM subsets (median [IQR] PP_robustness _= 0.642 [0.011–0.978]) and the most by matrix size (PP_robustness _= 0 [0–0]). Furthermore, every pairwise group comparison that included matrix size resulted in significantly different PP_robustness_ values (*p* = 0.009), and the same was true for all comparisons involving OSEM subsets (*p* = 0.009). When subjected to variations in other parameters specific to the OSEM algorithm, median PP_robustness_ was 0.075 [0.001–0.667] and 0.005 [0–0.103] for the number of iterations and updates, respectively.

Diminishing feature robustness was observed when comparing the effect of discordant z-axis filter kernels (PP_robustness _= 0.011 [0–0.213]) to isotropic Gaussian filter widths (PP_robustness _= 0.002 [0–0.036]), and to BPL β-values (PP_robustness _= 0.001 [0–0.026]). Neither changes in BPL β-value nor in the z-axis filter kernel produced significantly different PP_robustness_ values when compared to changes in Gaussian filter widths (*p* = 1 and 0.16, respectively). However, between BPL β-value and z-axis filter groups, differences in PP_robustness_ were themselves significant (*p* = 0.009).

For the remaining investigation groups, radiomic features exhibited significantly better stability to perturbations in acquisition time (PP_robustness_ = 0.072 [0–0.659]) than algorithm (PP_robustness_ = 0.0001 [0–0.004]) (*p* = 0.009). Comparisons with either of these parameter groups resulted in statistically significant different PP_robustness_ values with only a few exceptions: between acquisition time and number of OSEM iterations (*p* = 1) or z-axis filter kernel (*p* = 0.13), and between algorithm and BPL β-value (*p* = 0.13).

### 3.2. Correctable features

**Fig 4:**
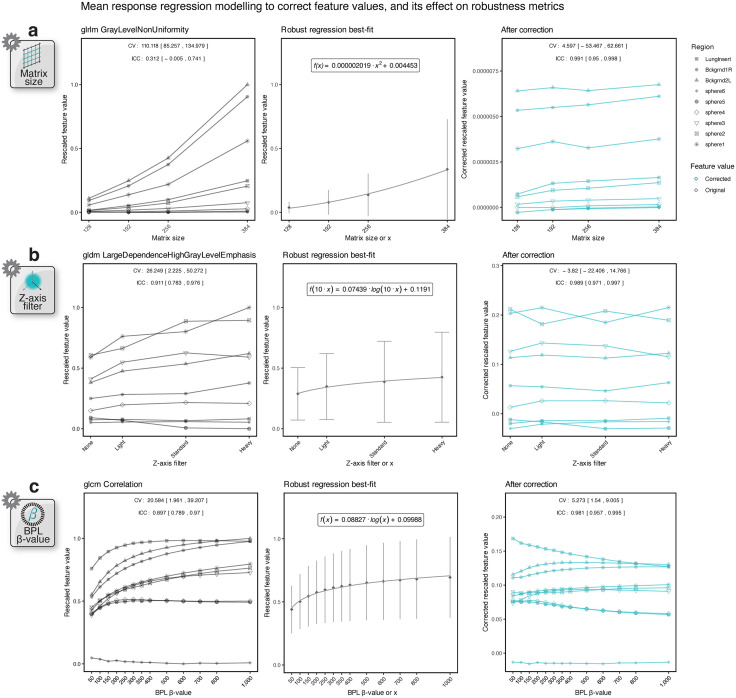
Correctability of radiomic features to variations in image acquisition and reconstruction settings. Feature correctability categorisations (a) and the predicted probabilities of producing correctable and moderately correctable features (PP_correctability_) per feature family **(b)**, both stratified by investigation group. Significance of differences in PP_correctability_ values between investigation groups **(c)**.

Correctability categorisations for non-robust features across investigation groups (barring algorithm) are presented in Fig 4a, with per-family feature proportions given in S3 Table. Scatter plots of the response of each feature against parameter variations after correction have been deposited in https://github.com/SyafiqRamlee/robust-radiomics-img-recon

**Fig 5:**
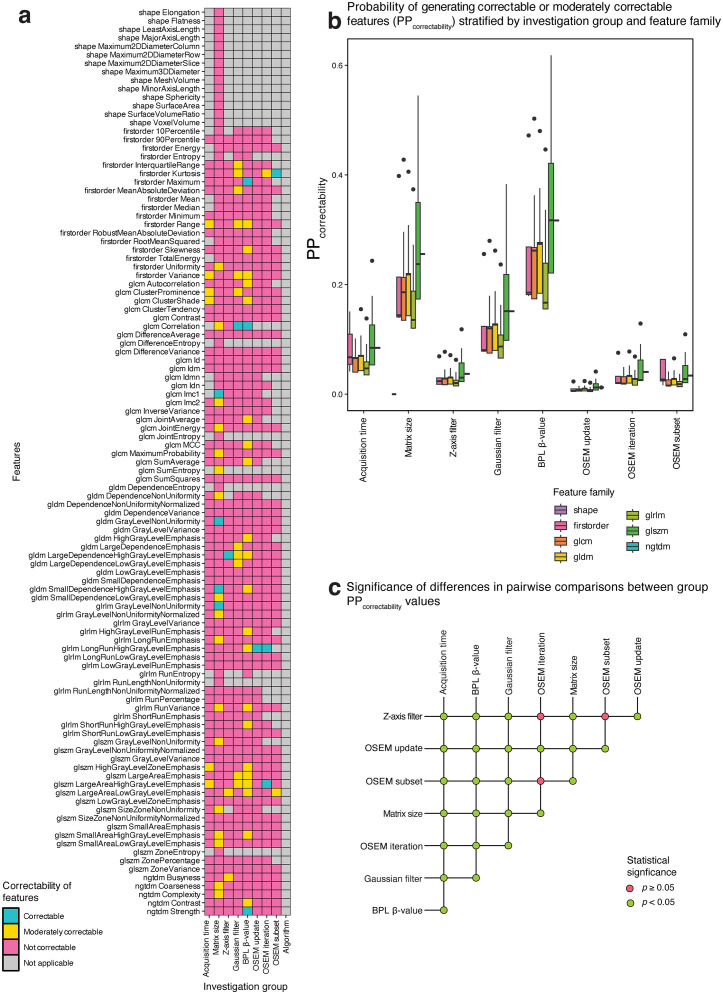
The effect of corrections using simple equations on three example radiomic features. Feature values tracked for every region for GLRLM *GrayLevelNonUniformity* **(a)**, GLDM *LargeDependenceHighGrayLevelEmphasis* **(b)**, and GLCM *Correlation* (c) against variations in matrix size, z-axis filter (kernel weight), and BPL β-value, respectively, are plotted on the left. Graphs showcasing the best-fit model describing the relationship of the corresponding mean feature values as a function of the reconstruction parameter are presented in the centre. Feature values corrected using the rearranged function of the best fit model are provided on the right. Uncorrected feature values have been rescaled using min-max normalisation.

Our analyses led us to discover only 13 correctable scenarios distributed across 11 radiomic features, as compiled in Table 2. Example graphs demonstrating the effect of correction for three of these instances are presented in Fig 5. The examples demonstrate the response to variations in transaxial matrix size for GLRLM *GrayLevelNonUniformity*, z-axis filter for GLDM *LargeDependenceHighGrayLevelEmphasis*, and BPL β-value for GLCM *Correlation*, respectively. In these instances, the effect of matrix size variations on the GLRLM feature values was observed to be best modelled by a quadratic function, whereas the dependence of the other two features on z-axis filter kernels or BPL β-values could be captured by a logarithmic equation. Applying corrections using the rearranged form of the models led to a reduction in *CV*_*mean*_ and improvement in ICC for these features, as annotated in Fig 5. The changes in *CV*_*mean*_ and ICC upon correction for the 13 non-robust features are presented as dumbbell plots in S1 Fig. Given that these radiomic features now meet the robust criteria following correction (*CV*mean, corrected**<10% and ICC*corrected*>0.90), they were deemed correctable.

**Table 2:** List of correctable feature scenarios.

Investigation group	Family	Feature
Matrix size	GLCM	*Imc1*
	GLDM	*GrayLevelNonUniformity*
		*SmallDependenceHighGrayLevelEmphasis*
	GLRLM	*GrayLevelNonUniformity*
Z-axis filter	GLDM	*LargeDependenceHighGrayLevelEmphasis*
Gaussian filter	GLCM	*Correlation*
BPL β-value	First-order	*Maximum*
	GLCM	*Correlation*
	NGTDM	*Strength*
OSEM updates	GLRLM	*LongRunHighGrayLevelEmphasis*
OSEM iterations	GLRLM	*LongRunHighGrayLevelEmphasis*
	GLSZM	*LargeAreaHighGrayLevelEmphasis*
OSEM subsets	First-order	*Kurtosis*

BPL: Bayesian penalised likelihood; OSEM: ordered subsets expectation maximisation; GLCM: grey-level co-occurrence matrix; GLDM: grey-level dependence matrix; GLRLM: grey-level run-length matrix; NGTDM: neighbouring grey-tone dependence matrix; GLSZM: grey-level size zone matrix; Imc1: Informational measure of correlation type 1.

Additionally, we identified 59 other scenarios in which features exhibited a reduction in *CV*_*mean*_ and increase in ICC after correction but failed to meet the robust criteria. These features were subsequently classified as moderately correctable. A list of these instances has been provided in S4 Table.

Fig 4b plots the predicted probability of generating correctable or moderately correctable features (PP_correctability_) for each investigation group. Results from pairwise comparisons between groups using the two-samples Cramér-von Mises test are presented in S5 Table and Fig 4c. Ranking the groups by median PP_correctability_, the order was as follows: BPL β-value (median [IQR] PP_correctability_ = 0.240 [0.181–0.317]), matrix size (PP_correctability_ = 0.173 [0.0135–0.219]), Gaussian filter (PP_correctability_ = 0.108 [0.079–0.151]), acquisition time (PP_correctability_ = 0.067 [0.043–0.084]), OSEM iterations (PP_correctability_ = 0.028 [0.02–0.041]), OSEM subsets (PP_correctability_ = 0.026 [0.017–0.034]), z-axis filter (PP_correctability_ = 0.026 [0.018–0.037]), and OSEM updates (PP_correctability_ = 0.008 [0.006–0.012]). Differences in PP_correctability_ values between groups achieved statistical significance (*p* = 0.007) for almost all pairwise comparisons. Exceptions to this were only observed between OSEM iterations, subsets, and z-axis filter groups (iterations vs. subsets, *p* = 1; vs. z-axis filter, *p* = 1; subsets vs. z-axis filter, *p* = 1).

### 3.3. Volume and intensity dependence of feature robustness

**Fig 6:**
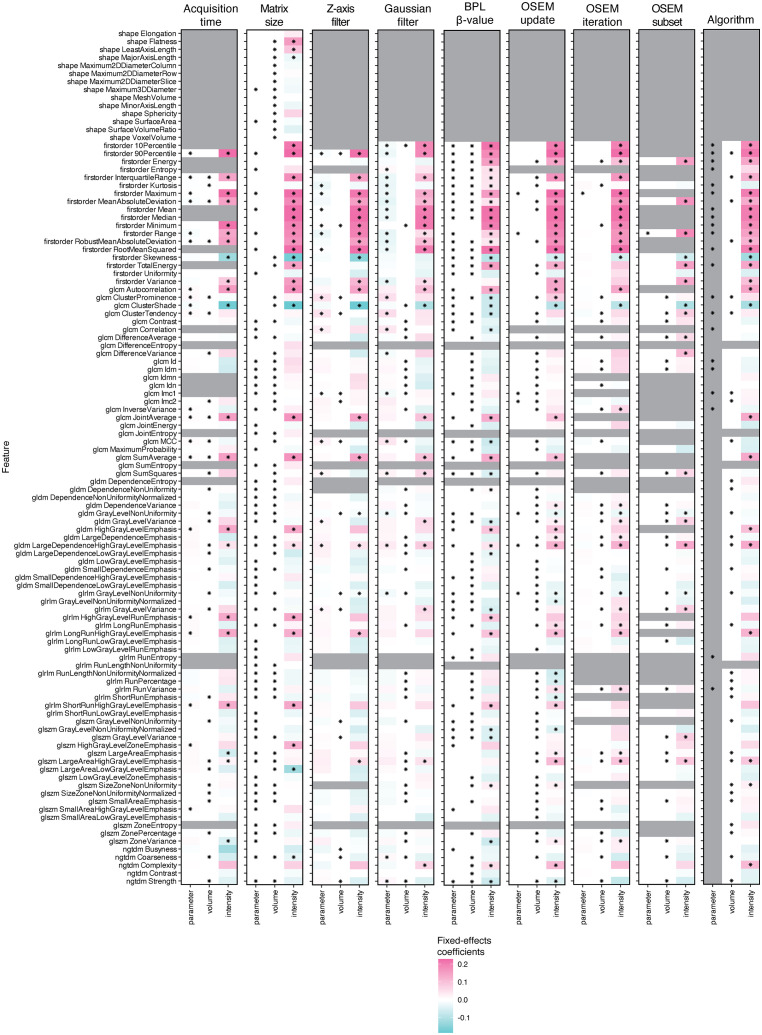
Analysis of the dependence of feature variability on region volume and intensity. Heatmaps of fixed-effects coefficients together with their statistical significance, i.e., *p* < 0.05 denoted by an asterisk (*), from linear mixed-effects models incorporating the reconstruction parameter under investigation, region volume, and intensity. We note that fixed-effects coefficients for the algorithm predictor have been greyed out given the categorical nature of the parameter.

In linear mixed-effects models, region volume, intensity, and the investigated acquisition/reconstruction parameter resulted in a differential effect on feature robustness, as illustrated in Fig 6. Irrespective of the investigation group, differences in region intensity generally exerted a stronger effect on feature robustness than region volume or acquisition/reconstruction parameter (as seen from the more conspicuous colours in Fig 6), with this effect skewing positive for most first-order features. The significance of these effects was also feature dependent.

**Fig 7:**
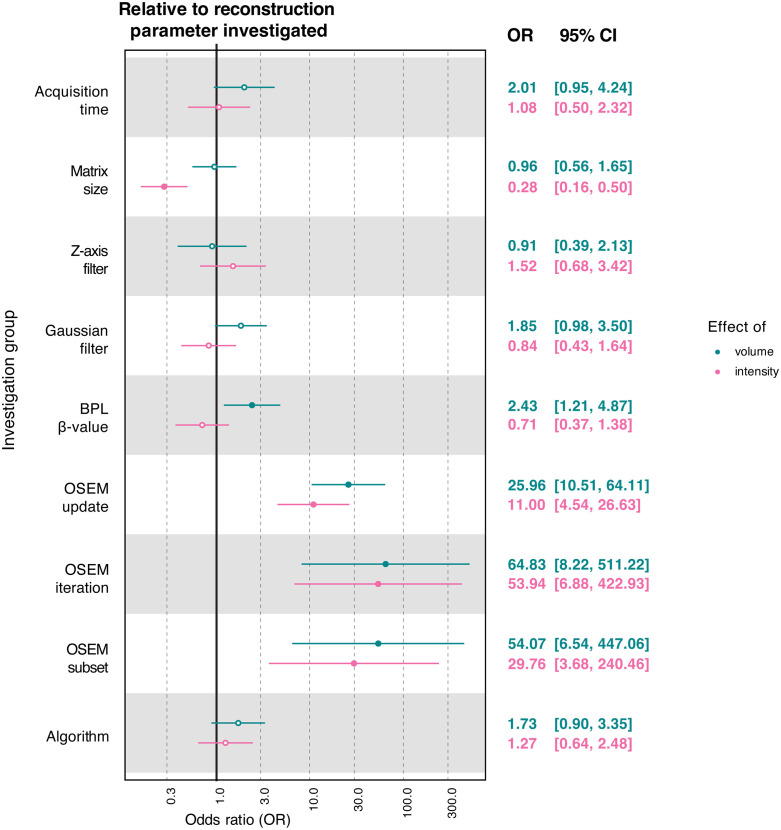
Effect of region volume and intensity on radiomic feature variability. Forest plots of the odds ratios (with 95% CI) for the effects of region volume or intensity on feature robustness, when compared to the effects of the image acquisition and reconstruction parameter under investigation. Non-significant results are displayed as hollow points.

Overall, region volume was a more likely determinant of feature robustness than the region intensity or acquisition/reconstruction parameter (Fig 7). Region volume particularly displayed a stronger tendency to affect feature robustness than variations in the number of OSEM iterations, subsets, updates, or BPL β-value, as evidenced by the odds ratios presented in Fig 7 (coloured in teal). Likewise, region intensity exhibited higher odds of substantially impacting feature robustness than OSEM updates, subsets, or iterations but these odds were lower for matrix size (Fig 7; coloured in pink).

## 4. Discussion

Radiomic features from [^18^F]-FDG-PET images could be used to support clinical decisions [3], but the formation of these images is reliant on a range of image acquisition and reconstruction parameters that can vary both within and between institutions. In a meta-analysis reviewing previous robustness studies involving PET radiomic features, image reconstruction parameters were found to impact feature robustness, although the strength of the supporting evidence was reported to be weak [43]. Ideally, radiomic features should reflect the characteristics of the region of interest (e.g., tumour lesion) alone, without exhibiting dependencies on such parameters [44,45]. This study examined the impact of different image acquisition/reconstruction settings on [^18^F]-FDG-PET radiomic features derived from the NEMA-IQ phantom, as a means to assess their stability in the absence of tumour image heterogeneity. We additionally investigated whether applying mathematical corrections to feature values could attenuate image acquisition/reconstruction effects, as previously explored in the context of image processing variations [36]. The effect of volume and intensity of interrogated regions on the robustness of feature values was also explored.

Our study revealed that the wide majority of [^18^F]-FDG-PET radiomic features were highly sensitive to changes in image acquisition or reconstruction settings irrespective of investigation group (acquisition time, matrix size, z-axis filter, Gaussian filter, BPL β-value, OSEM update, OSEM iteration, OSEM subset, and algorithm). Our results are therefore consistent with previous investigations [26,46,47], and reinforce the need for standardised imaging protocols or solutions to mitigate the effects of these parameters on feature robustness. Furthermore, we identified very few instances (i.e., 13 scenarios) in which features were correctable, indicating that most non-robust features did not exhibit a systematic dependency on acquisition/reconstruction parameters that could be modelled and corrected using simple equations. Some of the correctable features include the GLCM *Imc1* feature, which was not robust to matrix size variations but became robust following correction, suggesting that this feature could have been processed and used across [^18^F]-FDG PET images with different matrix sizes.

Our finding of a limited number of features correctable to variations in image acquisition and reconstruction parameters contrasts with a prior report wherein the dependencies of radiomic features on image processing parameters could be better mitigated through mathematical corrections [36]. This discrepancy suggests that parameter variations at the acquisition/reconstruction level merit greater attention when performing radiomics analyses. Alternative solutions, such as the batch effect corrections originally developed for genomics, called “ComBat” [48], and its downstream variants [49], could be required to correct radiomic measurements. In existing works, the ComBat approach has been demonstrated to be useful in harmonising features across image reconstruction parameters [48,50,51], all the more so given the difficulty in standardising acquisition/reconstruction parameters across different scanners, vendors, and centres [2]. Additionally, recent studies have utilised deep learning methods, such as the cycle-consistent generative adversarial networks (cycleGANs), to potentially synthesise more comparable images across scanners [49].

We found that the robustness of [^18^F]-FDG-PET radiomic features against variations in image reconstruction settings to be feature and family dependent. For instance, shape-based descriptors were only affected by matrix size whereas NGTDM features were affected by all the settings considered in this work. In a systematic review by Traverso et al., there is consensus that first-order *Entropy* is stable across image reconstruction settings in human and phantom PET studies [22,23,46,52]. In keeping with this observation, we noted entropy-related features were similarly robust: GLCM *DifferenceEntropy, JointEntropy, SumEntropy*, GLRLM *RunLengthNonUniformity, RunEntropy,* GLDM *DependenceEntropy*, and GLSZM *ZoneEntropy*. Several of these features were documented as stable in more recent reports [21,30,53,54], suggesting their suitability for radiomic evaluations across differently reconstructed PET images, such as in multi-centric studies.

Among the reconstruction parameters investigated, [^18^F]-FDG-PET feature values were the least robust to changes in transaxial image matrix size; an observation also shared by earlier publications [21,22]. One reason for this is that both the size and intensity values of voxels are affected by changes in this reconstruction parameter [55], especially when considering the partial volume effects inherent in PET images [56]. Despite this, matrix size ranked second in terms of generating correctable or moderately correctable features during our analysis, with the sensitivity of some features (e.g., GLRLM *GrayLevelNonUniformity*) mitigable through mathematical correction of feature values.

Choice of reconstruction algorithm induced strong effects on radiomic feature robustness. When considering OSEM, it is well known that image reconstructions with *n* iterations and *m* subsets are similar to *m* iterations and *n* subsets, and increasing either parameter—and especially both—results in elevated noise levels [53,57]. This is concordant with our results, where changes in the number of updates resulted in a low probability of achieving radiomic feature robustness. In the context of the BPL algorithm, perturbations in β-value led to even weaker feature robustness compared to changes in any of the OSEM parameters. This is also true when comparing variations in β-value against acquisition time, and concur with a recent investigation by Fooladi et al. who noted that β-value differences require more scrutiny during radiomics analyses than changes in acquisition duration [30].

Increasing BPL β-values, Gaussian filter widths, or z-axis filter kernel weights results in greater image smoothing, and we found their impact on radiomic features to be largely similar. Of the three, z-axis filtering was the most likely to produce robust features as it only affects smoothing along a single axis of the image. However, the correctability of radiomic features to these variations was significantly different between groups. This could be attributed to the differing number of data points available in each group for modelling (e.g., 21 for Gaussian filtering vs. 4 for z-axis filtering), which may have led to some differences in the efficacy of corrections.

Many radiomic features have a demonstrated dependency on volume [31–33], and it has also been shown in phantom PET studies that the size and intensity distribution of spheres affect feature robustness [26,29]. In agreement with this, we saw that the variability of [^18^F]-FDG-PET radiomic features was overall more likely to be significantly influenced by region volume or intensity than the acquisition/reconstruction parameter investigated. This helps explain why the feature corrections implemented in our work (which were based on the mean response of feature values across VOIs) may not have performed consistently across regions, as disparities in region volume or intensity can differentially affect the robustness of features. This is further substantiated by our finding that the investigation groups with the highest odds ratios when comparing region volume or intensity effects to the parameter under investigation (such as OSEM updates, iterations, and subsets) were ranked amongst the lowest in terms of feature correctability. Care should therefore be taken when pooling radiomic data from regions of interest with dissimilar volume and intensity characteristics.

This study bears several limitations. First, the results of this work were based on a phantom, which could be argued as being an oversimplified representation of actual tumours. However, by negating the biological variability found in tumours, our investigation enabled a controlled evaluation of the baseline stability of [^18^F]-FDG-PET radiomic features to image acquisition/reconstruction parameter variations. Additionally, the use of a uniform phantom helped minimise potential dependencies to radiomic extraction parameters. That being said, validation of our findings using clinical patient data, ideally obtained prospectively, and across different cancer types is warranted in future studies. Second, only eight functions were tested for feature corrections and the reliability of fits between groups of investigation may be impacted by the differing number of data points available for each group. A more extensive function library or a piecewise implementation could potentially improve model fits and correction. However, it should be noted that the use of more complex equations could increase the risk of overfitting and limit the generalisability of the correction approach. Third, future investigations may also explore higher-order features, together with the combined effect of reconstruction and other parameters (such as image processing parameters and segmentation) on radiomic features.

## 5. Conclusions

To conclude, phantom-derived [^18^F]-FDG-PET radiomic features were predominantly sensitive to variations in image reconstruction parameters, with robust features mainly composed of shape-based and entropy-related measurements. Most non-robust features did not exhibit a parameter dependency that could be addressed using simple mathematical corrections, and the robustness of these features was also shown to depend on the volume and intensity of analysed regions. These findings as a whole highlight the need for alternative solutions to mitigate the effects of discordant image reconstruction settings on feature robustness, and to ultimately exercise caution when handling radiomic data obtained from heterogeneously acquired/reconstructed [^18^F]-FDG-PET datasets.

## Supporting information

S1 Table The number of radiomic features (and percentage proportion out of the 107 features extracted) for each robustness category (NR: “not robust”; R: “robust”), segregated by feature family and investigation group. (PDF)

S2 Table Results from the two-sample test based on the Cramér-von Mises statistic comparing the PP_robustness_ values between investigation groups. *p*-values from post hoc analyses have been adjusted using the Bonferroni method. (PDF)

S3 Table The number of radiomic features (and percentage proportion out of the total number of features eligible for correction) for correctability categories (NC: “correctable”; MC: “moderately correctable”; C: “correctable”), segregated by feature family and investigation group. NA denotes “Not applicable”.
(PDF)

S1 Fig Dumbbell plots illustrating the change in CV and ICC upon correction for the 13 correctable feature scenarios identified in this work. Dashed lines represent thresholds of *CV* < 10% and ICC > 0.9.
(PDF)

S4 Table List of moderately correctable feature scenarios. (PDF)

S5 Table Results from the two-sample test based on the Cramér-von Mises statistic comparing the PP_correctability_ values between investigation groups. *p*-values from post hoc analyses have been adjusted using the Bonferroni method. (PDF)
