# Supplementary material for: On the robustness of [18F]-FDG-PET radiomic features to variations in image acquisition and reconstruction settings: A phantom study
Source: PLoS One. 2025 Oct 22;20(10):e0335219. doi: 10.1371/journal.pone.0335219 (PMC12543125; doi:10.1371/journal.pone.0335219)
Supplement: S1 Table — (PDF) [file pone.0335219.s001.pdf]

Table S1. The number of radiomic features (and percentage proportion out of the 107 features extracted) for each robustness category (NR: “not robust”; R: “robust”), segregated by feature family and investigation group.

	**Shape**		**First-order**		**GLCM**		**GLDM**		**GLRLM**		**GLSZM**		**NGTDM**	
	**NR**	**R**	**NR**	**R**	**NR**	**R**	**NR**	**R**	**NR**	**R**	**NR**	**R**	**NR**	**R**
Acquisition time	0 (0%)	14 (13%)	11 (10%)	7 (7%)	17 (16%)	7 (7%)	13 (12%)	1 (1%)	14 (13%)	2 (2%)	15 (14%)	1 (1%)	5 (5%)	0 (0%)
Matrix size	14 (13%)	0 (0%)	18 (17%)	0 (0%)	24 (22%)	0 (0%)	14 (13%)	0 (0%)	16 (15%)	0 (0%)	16 (15%)	0 (0%)	5 (5%)	0 (0%)
Z-axis filter	0 (0%)	14 (13%)	16 (15%)	2 (2%)	21 (20%)	3 (3%)	12 (11%)	2 (2%)	14 (13%)	2 (2%)	14 (13%)	2 (2%)	5 (5%)	0 (0%)
Gaussian filter	0 (0%)	14 (13%)	18 (17%)	0 (0%)	21 (20%)	3 (3%)	13 (12%)	1 (1%)	14 (13%)	2 (2%)	15 (14%)	1 (1%)	5 (5%)	0 (0%)
BPL β-value	0 (0%)	14 (13%)	18 (17%)	0 (0%)	21 (20%)	3 (3%)	13 (12%)	1 (1%)	15 (14%)	1 (1%)	15 (14%)	1 (1%)	5 (5%)	0 (0%)
OSEM updates	0 (0%)	14 (13%)	17 (16%)	1 (1%)	20 (19%)	4 (4%)	13 (12%)	1 (1%)	14 (13%)	2 (2%)	15 (14%)	1 (1%)	5 (5%)	0 (0%)
OSEM iterations	0 (0%)	14 (13%)	17 (16%)	1 (1%)	16 (15%)	8 (7%)	12 (11%)	2 (2%)	11 (10%)	5 (5%)	13 (12%)	3 (3%)	5 (5%)	0 (0%)
OSEM subsets	0 (0%)	14 (13%)	8 (7%)	10 (9%)	11 (10%)	13 (12%)	11 (10%)	3 (3%)	8 (7%)	8 (7%)	12 (11%)	4 (4%)	5 (5%)	0 (0%)
Algorithm	0 (0%)	14 (13%)	18 (17%)	0 (0%)	21 (20%)	3 (3%)	14 (13%)	0 (0%)	15 (14%)	1 (1%)	16 (15%)	0 (0%)	5 (5%)	0 (0%)
