# Supplementary material for: On the robustness of [18F]-FDG-PET radiomic features to variations in image acquisition and reconstruction settings: A phantom study
Source: PLoS One. 2025 Oct 22;20(10):e0335219. doi: 10.1371/journal.pone.0335219 (PMC12543125; doi:10.1371/journal.pone.0335219)
Supplement: S2 Table — (PDF) [file pone.0335219.s002.pdf]

Table S2. Results from the two-sample test based on the Cramér-von Mises statistic comparing the $PP_{robustness}$ values between investigation groups. *p*-values from post hoc analyses have been adjusted using the Bonferroni method.

**$Group_1$**	**$Group_2$**	**Test statistic**	**Adjusted *p*-value**
Acquisition time	Algorithm	20.4	**0.009**
Acquisition time	BPL β-value	11.4	**0.009**
Acquisition time	Gaussian filter	11.28	**0.009**
Acquisition time	OSEM iterations	0.75	1
Acquisition time	Matrix size	68.94	**0.009**
Acquisition time	OSEM subsets	8.54	**0.009**
Acquisition time	OSEM updates	8.02	**0.009**
Acquisition time	Z-axis filter	4.05	0.126
Matrix size	OSEM subsets	68.94	**0.009**
Matrix size	OSEM updates	59.47	**0.009**
Matrix size	Z-axis filter	59.47	**0.009**
Gaussian filter	OSEM iterations	11.28	**0.009**
Gaussian filter	Matrix size	52.04	**0.009**
Gaussian filter	OSEM subsets	22.08	**0.009**
Gaussian filter	OSEM updates	2.42	0.954
Gaussian filter	Z-axis filter	4.05	0.162
BPL β-value	Gaussian filter	1.86	1
BPL β-value	OSEM iterations	11.4	**0.009**
BPL β-value	Matrix size	52.04	**0.009**
BPL β-value	OSEM subsets	26.58	**0.009**
BPL β-value	OSEM updates	2.51	0.774
BPL β-value	Z-axis filter	6.38	**0.009**
OSEM updates	Z-axis filter	2.4	1
OSEM iterations	Matrix size	68.94	**0.009**
OSEM iterations	OSEM subsets	8.48	**0.009**
OSEM iterations	OSEM updates	8.11	**0.009**
OSEM iterations	Z-axis filter	4.44	0.072
OSEM subsets	OSEM updates	19.97	**0.009**
OSEM subsets	Z-axis filter	12.73	**0.009**
Algorithm	BPL β-value	4.07	0.126
Algorithm	Gaussian filter	6.4	**0.009**
Algorithm	OSEM iterations	20.63	**0.009**
Algorithm	Matrix size	51.96	**0.009**
Algorithm	OSEM subsets	30.67	**0.009**
Algorithm	OSEM updates	9.6	**0.009**
Algorithm	Z-axis filter	11.46	**0.009**
