# Supplementary material for: On the robustness of [18F]-FDG-PET radiomic features to variations in image acquisition and reconstruction settings: A phantom study
Source: PLoS One. 2025 Oct 22;20(10):e0335219. doi: 10.1371/journal.pone.0335219 (PMC12543125; doi:10.1371/journal.pone.0335219)
Supplement: S3 Table — NA denotes “Not applicable”. (PDF) [file pone.0335219.s003.pdf]

Table S3. The number of radiomic features (and percentage proportion out of the total number of features eligible for correction) for correctability categories (NC: “correctable”; MC: “moderately correctable”; C: “correctable”), segregated by feature family and investigation group. NA denotes “Not applicable”.

	**Shape**	**First-order**			**GLCM**			**GLDM**			**GLRLM**			**GLSZM**			**NGTDM**		
	**NC**	**NC**	**MC**	**C**	**NC**	**MC**	**C**	**NC**	**MC**	**C**	**NC**	**MC**	**C**	**NC**	**MC**	**C**	**NC**	**MC**	**C**
Acquisition time	0 (0%)	9 (12%)	2 (3%)	0 (0%)	15 (20%)	2 (3%)	0 (0%)	13 (17%)	0 (0%)	0 (0%)	14 (19%)	0 (0%)	0 (0%)	13 (17%)	2 (3%)	0 (0%)	5 (7%)	0 (0%)	0 (0%)
Matrix size	14 (13%)	17 (16%)	1 (1%)	0 (0%)	18 (17%)	5 (5%)	1 (1%)	10 (9%)	2 (2%)	2 (2%)	12 (11%)	3 (3%)	1 (1%)	12 (11%)	4 (4%)	0 (0%)	3 (3%)	2 (2%)	0 (0%)
Z-axis filter	0 (0%)	16 (20%)	0 (0%)	0 (0%)	21 (26%)	0 (0%)	0 (0%)	11 (13%)	0 (0%)	1 (1%)	14 (17%)	0 (0%)	0 (0%)	13 (16%)	1 (1%)	0 (0%)	4 (5%)	1 (1%)	0 (0%)
Gaussian filter	0 (0%)	13 (15%)	5 (6%)	0 (0%)	19 (22%)	1 (1%)	1 (1%)	10 (12%)	3 (3%)	0 (0%)	14 (16%)	0 (0%)	0 (0%)	13 (15%)	2 (2%)	0 (0%)	5 (6%)	0 (0%)	0 (0%)
BPL β-value	0 (0%)	14 (16%)	3 (3%)	1 (1%)	15 (17%)	5 (6%)	1 (1%)	10 (11%)	3 (3%)	0 (0%)	11 (13%)	4 (5%)	0 (0%)	10 (11%)	5 (6%)	0 (0%)	3 (3%)	1 (1%)	1 (1%)
OSEM updates	0 (0%)	17 (20%)	0 (0%)	0 (0%)	20 (24%)	0 (0%)	0 (0%)	13 (15%)	0 (0%)	0 (0%)	13 (15%)	0 (0%)	1 (1%)	15 (18%)	0 (0%)	0 (0%)	5 (6%)	0 (0%)	0 (0%)
OSEM iterations	0 (0%)	16 (22%)	1 (1%)	0 (0%)	16 (22%)	0 (0%)	0 (0%)	12 (16%)	0 (0%)	0 (0%)	10 (14%)	0 (0%)	1 (1%)	12 (16%)	0 (0%)	1 (1%)	5 (7%)	0 (0%)	0 (0%)
OSEM subsets	0 (0%)	7 (13%)	0 (0%)	1 (2%)	11 (20%)	0 (0%)	0 (0%)	11 (20%)	0 (0%)	0 (0%)	8 (15%)	0 (0%)	0 (0%)	11 (20%)	1 (2%)	0 (0%)	5 (9%)	0 (0%)	0 (0%)
Algorithm	NA	NA	NA	NA	NA	NA	NA	NA	NA	NA	NA	NA	NA	NA	NA	NA	NA	NA	NA
