# Supplementary material for: On the robustness of [18F]-FDG-PET radiomic features to variations in image acquisition and reconstruction settings: A phantom study
Source: PLoS One. 2025 Oct 22;20(10):e0335219. doi: 10.1371/journal.pone.0335219 (PMC12543125; doi:10.1371/journal.pone.0335219)
Supplement: S1 Fig — Dashed lines represent thresholds of CV < 10% and ICC > 0.9. (PDF) [file pone.0335219.s004.pdf]

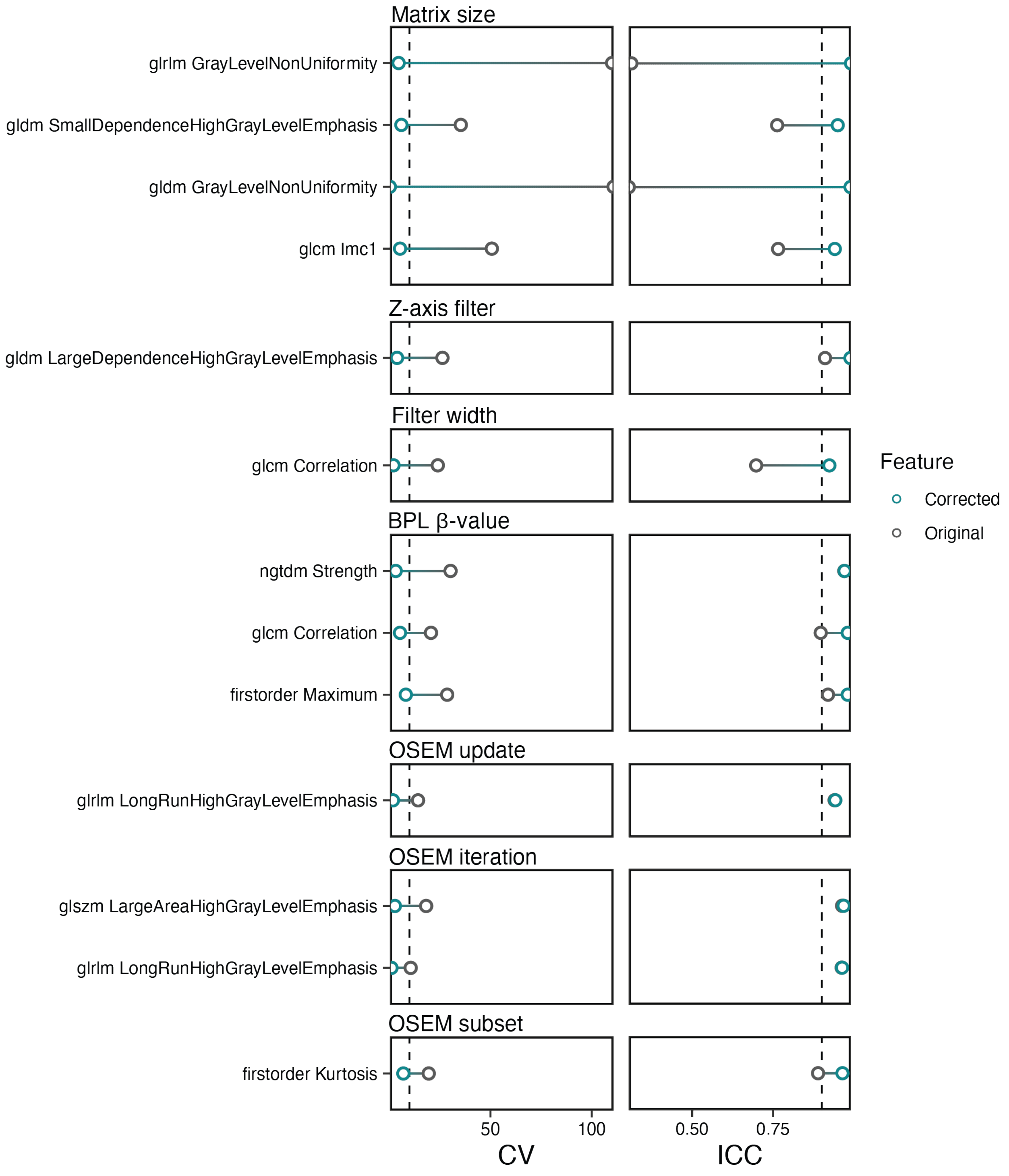

Figure S1. Dumbbell plots illustrating the change in CV and ICC upon correction for the 13 correctable feature scenarios identified in this work. Dashed lines represent thresholds of *CV* < 10% and ICC > 0.9.
