# Supplementary material for: On the robustness of [18F]-FDG-PET radiomic features to variations in image acquisition and reconstruction settings: A phantom study
Source: PLoS One. 2025 Oct 22;20(10):e0335219. doi: 10.1371/journal.pone.0335219 (PMC12543125; doi:10.1371/journal.pone.0335219)
Supplement: S4 Table — (PDF) [file pone.0335219.s005.pdf]

Table S4. List of moderately correctable feature scenarios.

**Investigation group**	**Family**	**Feature**
Acquisition time	First-order	*Range*
		Variance
	GLCM	*ClusterProminence*
		ClusterShade
	GLSZM	*HighGrayLevelZoneEmphasis*
		LargeAreaHighGrayLevelEmphasis
Matrix size	First-order	*Uniformity*
	GLCM	*Correlation*
		Imc2
		JointEnergy
		MaximumProbability
		SumEntropy
	GLDM	*DependenceNonUniformity*
		SmallDependenceLowGrayLevelEmphasis
	GLRLM	*GrayLevelNonUniformityNormalized*
		LongRunEmphasis
		RunVariance
	GLSZM	*GrayLevelNonUniformity*
		SizeZoneNonUniformity
		SmallAreaHighGrayLevelEmphasis
		SmallAreaLowGrayLevelEmphasis
	NGTDM	*Coarseness*
		Complexity
Z-axis filter	GLSZM	*LargeAreaLowGrayLevelEmphasis*
	NGTDM	*Busyness*
Gaussian filter	First-order	*InterquartileRange*
		Kurtosis
		MeanAbsoluteDeviation
		Range
		Variance
	GLCM	*ClusterProminence*
	GLDM	*LargeDependenceEmphasis*
		LargeDependenceHighGrayLevelEmphasis
		LargeDependenceLowGrayLevelEmphasis
	GLSZM	*LargeAreaEmphasis*
		LargeAreaHighGrayLevelEmphasis
BPL β-value	First-order	*Range*
		Skewness
		Variance
	GLCM	*Autocorrelation*
		ClusterShade
		JointAverage
		MCC
		SumAverage

	GLDM	*HighGrayLevelEmphasis*
		LargeDependenceHighGrayLevelEmphasis
		SmallDependenceHighGrayLevelEmphasis
	GLRLM	*HighGrayLevelRunEmphasis*
		LongRunHighGrayLevelEmphasis
		RunVariance
		ShortRunHighGrayLevelEmphasis
	GLSZM	*HighGrayLevelZoneEmphasis*
		LargeAreaEmphasis
		LargeAreaHighGrayLevelEmphasis
		LargeAreaLowGrayLevelEmphasis
		SmallAreaHighGrayLevelEmphasis
	NGTDM	*Contrast*
OSEM iterations	First-order	*Kurtosis*
OSEM subsets	GLSZM	*LargeAreaLowGrayLevelEmphasis*
