# Supplementary material for: On the robustness of [18F]-FDG-PET radiomic features to variations in image acquisition and reconstruction settings: A phantom study
Source: PLoS One. 2025 Oct 22;20(10):e0335219. doi: 10.1371/journal.pone.0335219 (PMC12543125; doi:10.1371/journal.pone.0335219)
Supplement: S5 Table — (PDF) [file pone.0335219.s006.pdf]

Table S5. Results from the two-sample test based on the Cramér-von Mises statistic comparing the $PP_{correctability}$ values between investigation groups. *p*-values from post hoc analyses have been adjusted using the Bonferroni method.

$Group_1$	$Group_2$	Test statistic	Adjusted *p*-value
Acquisition time	BPL β-value	50.86	**0.007**
Acquisition time	Gaussian filter	20.27	**0.007**
Acquisition time	OSEM iterations	26.98	**0.007**
Acquisition time	Matrix size	31.55	**0.007**
Acquisition time	OSEM subsets	27.06	**0.007**
Acquisition time	OSEM updates	51.82	**0.007**
Acquisition time	Z-axis filter	31.53	**0.007**
Matrix size	OSEM subsets	35.56	**0.007**
Matrix size	OSEM updates	41.79	**0.007**
Matrix size	Z-axis filter	41.32	**0.007**
Gaussian filter	OSEM iterations	46.73	**0.007**
Gaussian filter	Matrix size	14.92	**0.007**
Gaussian filter	OSEM subsets	44.15	**0.007**
Gaussian filter	OSEM updates	56.29	**0.007**
Gaussian filter	Z-axis filter	51	**0.007**
BPL β-value	Gaussian filter	38.61	**0.007**
BPL β-value	OSEM iterations	53.33	**0.007**
BPL β-value	Matrix size	13.88	**0.007**
BPL β-value	OSEM subsets	47.19	**0.007**
BPL β-value	OSEM updates	56.65	**0.007**
BPL β-value	Z-axis filter	55.94	**0.007**
OSEM updates	Z-axis filter	42.08	**0.007**
OSEM iterations	Matrix size	39.34	**0.007**
OSEM iterations	OSEM subsets	1.5	1
OSEM iterations	OSEM updates	42.42	**0.007**
OSEM iterations	Z-axis filter	2.21	1
OSEM subsets	OSEM updates	34.01	**0.007**
OSEM subsets	Z-axis filter	0.93	1
